# Occurrence of long-finned pilot whales (*Globicephala melas*) and killer whales (*Orcinus orca*) in Icelandic coastal waters and their interspecific interactions

**DOI:** 10.1007/s10211-022-00394-1

**Published:** 2022-06-07

**Authors:** Anna Selbmann, Charla J. Basran, Chiara G. Bertulli, Tess Hudson, Marie-Thérèse Mrusczok, Marianne H. Rasmussen, Jonathan N. Rempel, Judith Scott, Jörundur Svavarsson, Paul J. Wensveen, Megan Whittaker, Filipa I. P. Samarra

**Affiliations:** 1 Faculty of Life and Environmental Sciences, University of Iceland, Sturlugata 7, 101 Reykjavík, Iceland; 2 University of Iceland’s Research Center, Húsavík, Iceland; 3 Sea Watch Foundation, Amlwch, UK; 4 Whale Watching Akureyri, Akureyri, Iceland; 5 Orca Guardians Iceland, Grundarfjörður, Iceland; 6 Láki Tours, Grundarfjörður, Iceland; 7 Náttúrustofa Vesturlands, Stykkishólmur, Iceland; 8 Special Tours, Reykjavík, Iceland; 9 Elding Whale Watching, Reykjavík, Iceland; 10 University of Iceland’s Institute of Research Centres, Vestmannaeyjar, Iceland

**Keywords:** Interspecific interactions, Antagonism, Distribution shifts, Opportunistic data

## Abstract

Long-finned pilot whales and killer whales are widely distributed across the North Atlantic, but few studies have reported their occurrence in Icelandic coastal waters. Here, we use sightings data from research platforms and whale watching tours in six regions of Iceland from 2007 to 2020 to show that the occurrence of long-finned pilot and killer whales varied with region and season. Killer whales were regularly encountered in the south of Iceland during summer and west of Iceland during winter/spring. Long-finned pilot whales were only seen during the summer and were most often encountered in the south, west, and northwest of Iceland. Long-finned pilot whale occurrence in the south of Iceland appeared to increase during the study period but killer whale occurrence showed no noticeable changes. Long-finned pilot whales were sighted often in the areas that were also frequented by killer whales and interspecific interactions were commonly observed when both species co-occurred. Interactions appeared to be antagonistic, with killer whales often avoiding long-finned pilot whales and sometimes fleeing at high speed, similar to what has been described elsewhere in the North Atlantic. In the majority of interactions observed (68%), killer whales avoided long-finned pilot whales by moving away, but in 28% avoidance was at high speed with both species porpoising. This variability in the type of behavioural responses indicates that interactions may be more complex than previously described. We discuss regional trends in long-finned pilot whale and killer whale sightings and potential drivers of the observed interactions.

**Supplementary Information:**

The online version contains supplementary material available at 10.1007/s10211-022-00394-1.

## Introduction

Pilot whales and killer whales are widely distributed in the world’s oceans (Ford 2018; Olson 2018), and in the case of pilot whales, two different species are recognised, which have only some areas where occurrence overlaps: the short-finned (*Globicephala macrorhynchus*) and long-finned pilot whale (*Globicephala melas*). The first has a pantropical and pan-temperate distribution, whereas the latter is found in cold temperate to sub-polar waters of the North Atlantic and the Southern hemisphere. The long-finned pilot whale (hereafter, pilot whale) has two recognised extant subspecies separated by a wide tropical belt: *G. m. melas* and *G. m. edwardii* (Olson 2018). Pilot whales overlap with killer whales in their geographical range in the North Atlantic and Southern Ocean (Ford 2018; Olson 2018); however, the two species appear to have different habitat preferences. Killer whales more commonly occur in shallow coastal waters, while pilot whales prefer deep-water habitats on the continental shelf break and slopes but often move seasonally between coastal and offshore waters (Ford 2018; Olson 2018).

In the Northeast Atlantic, line-transect surveys conducted in the summer since 1987 indicate a widespread offshore distribution of pilot whales (Buckland et al. 1993; Pike et al. 2019). Around Iceland, high occurrence is reported in the southwest and along the continental shelf edge in the south, but not in the north and northeast (Buckland et al. 1993; Pike et al. 2019). However, the occurrence of the species in coastal waters is irregular and thus its distribution and occurrence patterns are understudied. Pilot whale mass strandings occur in Iceland occasionally (Sigurjónsson et al. 1993), and Sæmundsson (1937, 1939) suggested that these could be linked to changes in the migration of their squid prey into Icelandic coastal waters. However, little is known of the diet of pilot whales occurring in this region and the only information comes from four animals stranded in 1982 that contained only beaks of flying squid (*Todarodes sagittatus*) in their stomachs (Sigurjónsson et al. 1993).

Killer whales are encountered offshore in the Northeast Atlantic but encounter rates tend to be low and irregular (Pike et al. 2020). Thus, the precision of abundance estimates from line-transect surveys is low and population trends remain unclear (Foote et al. 2007; Pike et al. 2020). While sightings occur all around Iceland, highest densities are reported to the east and northeast (Pike et al. 2020). In certain coastal areas around Iceland on the other hand, killer whales are known to occur commonly and regularly, particularly in areas where herring (*Clupea harengus*) gathers to spawn or overwinter (Sigurjónsson et al. 1988; Samarra et al. 2017). A large part of the population appears to feed on herring, following the Icelandic summer-spawning herring migration between their spawning grounds around the Vestmannaeyjar archipelago in the south and their wintering grounds in Breiðafjörður in the west of Iceland (Samarra et al. 2017). However, these are also the regions where most research efforts have focused on, and so, little is known of the occurrence of killer whales in other coastal regions.

In Norway and the Strait of Gibraltar (Spain), where killer whales and pilot whales regularly co-occur in coastal waters, interactions between both species have been observed (Stenersen and Similä 2004; de Stephanis et al. 2014). In all of the interactions reported, the pilot whales moved towards the killer whales which caused the killer whales to move directly away, sometimes at high speed (Stenersen and Similä 2004; de Stephanis et al. 2014). Playback experiments simulating killer whale presence showed that both short- and long-finned pilot whales are strongly attracted to killer whale sounds (Curé et al. 2012, 2019; Bowers et al. 2018), unlike other marine mammal species in similar experiments (e.g. Deecke et al. 2002; Curé et al. 2015). Two main hypotheses for the observed antagonistic interactions between pilot whales and killer whales have been brought forward: the interactions could be driven by food competition or could be a mobbing strategy based on anti-predator behaviour (Stenersen and Similä 2004; Curé et al. 2012; de Stephanis et al. 2014). However, much remains unclear as to why these interactions occur, what their relevance could be, and what the consequences are to both species. Possible impacts of such interactions are likely higher if interactions occur regularly due to spatial and temporal overlap.

Here, we investigated the occurrence of pilot whales and killer whales in different coastal regions of Iceland to assess the spatial and temporal overlap between both species. Then, we provide the first detailed descriptions of observed interactions between the two species in Iceland, discussing their frequency, the types of behavioural responses observed, and their potential causes and consequences.

## Methods

**Fig. 1:**
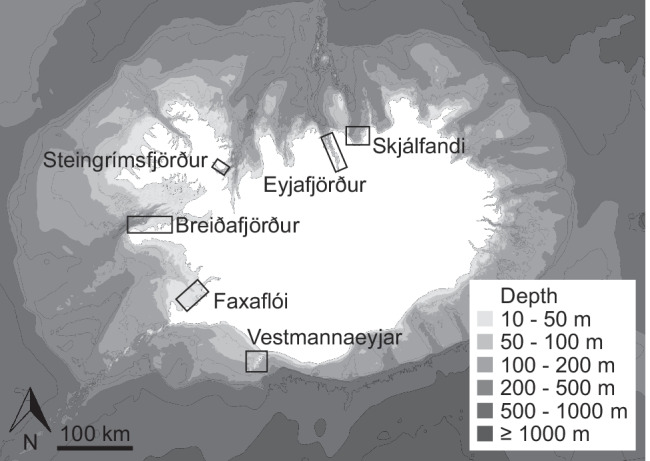
Map of Iceland indicating study areas and bathymetry. Bathymetric data: Icelandic Coast Guard, Hydrographic Department

Data were collected from 2007 to 2020 in six areas around Iceland (Fig. 1). Effort and sightings data came from dedicated research (Vestmannaeyjar) or opportunistic data collection by researchers and naturalists onboard whale watching tours (all other locations). Whale watching tours were operated on different types of boats (12–34 m length) but generally included lookouts a few metres above sea level. At least one observer was actively searching for whales and taking notes on species sighted. In Faxaflói, Breiðafjörður, and Eyjafjörður boats departed from different harbours but generally search areas varied little and thus were considered as one region. The boat-based research in Vestmannaeyjar was conducted from small boats (6–8 m length) with at least one dedicated observer onboard, who either took notes on paper or recorded audio notes. Research focus varied between years but notes were generally taken on the species sighted, including estimates of group size and behaviour, with particular attention to feeding behaviours and prey species. In Vestmannaeyjar, visual surveys were also undertaken from a vantage point on land (2017–2020), located at 100 m height at the southern tip of Heimaey, the main island of the Vestmannaeyjar archipelago. Several small islands obstruct the view in some areas but the location generally provides a good overview of the study area. Typically, 2–5 observers conducted regular scan surveys, as well as periods of opportunistic search, throughout the day using 7 × 50 (Steiner Navigator pro) and 15 × 70 (Helios Stellar-II) binoculars. All marine mammal species sighted were recorded, including estimates of group size and behaviour. A calibrated digital theodolite (Sokkia DT-510) was used with the software VADAR (Visual and Acoustic Detection and Ranging, E. Kniest, University of Newcastle) to record positions of sightings and track groups of killer whales and/or pilot whales whenever possible.

Survey effort varied between seasons and locations, with the summer months being covered more extensively due to longer daylight hours and higher tourism activity (up to six whale watching tours per day in some locations, approximately 3 h each). The winter daylight hours often only allowed for one whale watching tour per day. In 2020, effort across all locations was much reduced due to the COVID-19 pandemic. Certain individuals or groups may avoid or approach boats more than others, which can lead to a bias in individuals observed but due to the opportunistic nature of the data collection, it was not possible to control for such potential biases.

Occurrence of pilot and killer whales was calculated as the percentage of days with effort that included sightings of either species. Changes in occurrence over time were investigated using yearly total percentages of occurrence for each location and using Pearson’s correlation or Spearman’s rank tests if data were not normally distributed based on a Shapiro–Wilk test (significance level 0.05).

Observations of interactions between pilot whales and killer whales were collected ad libitum (Mann 1999). An interaction was defined as either of the two species approaching the other and the approach possibly resulting in a change in behaviour of the second species. The start and end of an interaction were defined as the first and last concurrent sightings of both species. The end could have several reasons: either species could move out of the area or could disappear from view behind an island, or in the case of observations from a boat, the boat could leave the area. Group sizes during interactions were generally recorded by the observers as best estimates but sometimes as ranges or minimum estimates, in which case the mean or minimum values were used in the analysis (e.g. 25 for a recorded 20–30 individuals and 50 for a recorded 50 + individuals). If several groups were seen, group sizes could refer to the size of the group involved in the interaction or the total of individuals in the area. Only in a few cases were such differences clearly noted and thus further analysis was conducted on group sizes of the total individuals seen. In Vestmannaeyjar, the same interaction could sometimes be observed from the boat and land. In these cases, only observations from land were analysed, as observers from land generally had a better overview of the interactions and groups of whales in the area.

## Results

### Occurrence of pilot and killer whales

In total, 8090 days of effort were recorded from 2007 to 2020 across all locations, including 183 days (2.3%) with pilot whale and 647 days (8.0%) with killer whale sightings. However, effort and occurrence of the two species varied considerably between sites (Table 1).

**Table 1:** Occurrence of pilot whales (PW) and killer whales (KW), their interactions, and the total survey effort in different locations around Iceland, 2007–2020. Occurrence and interactions are given as percentage of effort days, with the total number of days in brackets. ‘Co-occurrence’ refers to days with sightings of both pilot whales and killer whales, but where both species were not necessarily sighted at the same time

Region	Location	Occurrence PW % (days)	Occurrence KW % (days)	Co-occurrence % (days)	Interactions % (days)	Effort days
Northeast	Skjálfandi	0.1 (2)	1.1 (21)	0	0	1928
Northeast	Eyjafjörður	0.7 (8)	0.7 (8)	0	0	1146
Northwest	Steingrímsfjörður	15.8 (37)	3.4 (8)	0	0	234
West	Breiðafjörður	8.3 (84)	38.1 (387)	0.7 (7)	0.4 (4)	1017
Southwest	Faxaflói	0.4 (14)	1.5 (55)	0	0	3552
South	Vestmannaeyjar	17.8 (38)	80.3 (171)	17.4 (37)	9.4 (20)	213

**Fig. 2:**
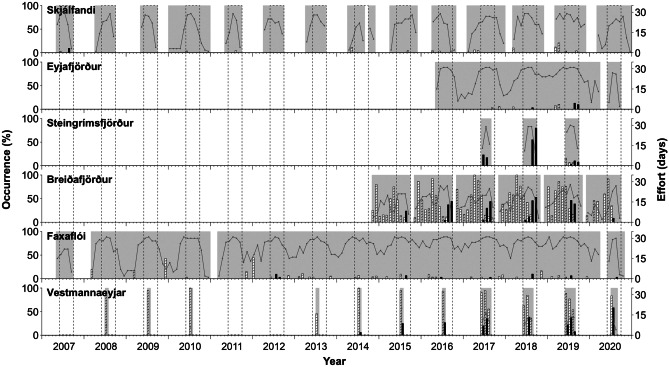
Monthly occurrence of pilot whales (black bars) and killer whales (hatched bars) at different locations in Icelandic coastal waters. Periods with effort highlighted in grey with number of days of effort per month as a line, and summer periods (June–September) indicated with dashed boxes

Pilot whales were only encountered from June to September (Fig. 2). Pilot whale occurrence increased over the study period in four out of six locations (all except Steingrímsfjörður and Skjálfandi), but its correlation with year was statistically significant only in Vestmannaeyjar (r_(9)_ = 0.85, *p* < 0.01) and Faxaflói (r_s_ = 0.69, *p* < 0.01). Pilot whales were sighted most regularly in Vestmannaeyjar, Breiðafjörður, and Steingrímsfjörður. In all other locations, sightings were irregular and infrequent (Table 1 and Fig. 2).

Killer whales were encountered year-round but occurrence varied between seasons and locations (Fig. 2). They were sighted regularly in the summer months in Vestmannaeyjar. In Breiðafjörður, occurrence was highest in winter and spring. In all other locations, killer whale encounters were infrequent and there was no clear seasonal pattern in their occurrence. There was no significant positive or negative correlation in occurrence of killer whales with year for any location. In the two areas with the highest occurrence of killer whales, Vestmannaeyjar and Breiðafjörður, occurrence of pilot whales was also high (Table 1 and Fig. 2). These were also the only two locations where both species were sighted on the same days and behavioural interactions between the two species were observed (Table 1).

### Interactions between pilot and killer whales

Although both killer whales and pilot whales occurred in the same area on 44 days of effort (Table 1), some of these sightings occurred at different times of the day such that both species were not simultaneously present. In 34 of these 44 days, pilot and killer whales were observed simultaneously, providing the potential for interactions to occur (Table 2). Interactions were observed on 70.6% of these days, or on a total of 24 days, 20 in Vestmannaeyjar and four in Breiðafjörður. The difference between these two locations may be due to a greater temporal overlap of the two species in Vestmannaeyjar, where both were common in summer. In contrast, in Breiðafjörður pilot whales were mostly seen in summer and killer whales were most common in winter and spring (Fig. 2). The mean duration of the observed interactions was 62 ± 71 min (mean ± standard deviation; range 6–286). The mean number of killer whales and pilot whales involved in the interactions was 27 ± 27 individuals (range 4–100) and 52 ± 39 individuals (range 20–150), respectively.

**Table 2:** Summarised simultaneous observations of pilot whales (PW) and killer whales (KW) in Iceland, and interactions between them. Number of individuals refers to the total number of animals observed. If several groups were seen, not all of them always responded in the same manner and details on the number of individuals involved in the interactions are given in the comments

Location	Date	Observation platform	Interaction duration (min)	KW number	PW number	KW response	Comments
Vestmannaeyjar	2014–07–06	Boat	NA	~100	NA	Avoidance	PW approach at high speed, KW move away
	2015–07–11	Boat	58	NA	NA	Avoidance	PW approach twice, KW move away
	2015–07–13	Boat	6	NA	NA	No response, avoidance	Two groups of KW. PW approach fast and one group of KW moves away, the other stays and continues feeding
	2015–07–17	Boat	69	NA	NA	Avoidance	Two groups of KW. PW are seen several times and once clearly approach the KW and the KW move away
	2015–07–18	Boat	168	NA	NA	Avoidance	PW approach KW at high speed. KW are not seen for ~ 30 min but are then sighted again further away. PW approach again and KW move away
	2016–07–20	Boat	130	NA	NA	Avoidance	PW approach KW at high speed twice, KW move away
	2016–07–21	Boat	37	NA	NA	High-speed avoidance	PW approach at high speed, KW move away at high speed (porpoising)
	2016–07–22	Boat	NA	NA	NA	Avoidance	PW approach KW, KW move away. KW described as ‘fleeing’ but porpoising not confirmed. Possible but unconfirmed high-speed avoidance
	2016–07–27	Boat	NA	NA	NA	NA	PW approach KW at high speed but not known how KW respond
	2017–06–18	Land	6	4–5	NA	High-speed avoidance	PW approach KW at high speed, KW move away at high speed (porpoising)
	2017–06–19	Land	NA	4+	10–30+	Avoidance	PW approach KW, KW move away. KW described as being ‘chased’ by PW but porpoising not confirmed. Possible but unconfirmed high-speed avoidance
		Boat	NA	12	~50	NA	KW observed but sight of them lost for ~ 20 min. They are resighted after PW passed. Likely the same interaction as observed from land
	2017–07–04	Land	NA	1+	9+	NA	One sighting of a KW with a group of PW right behind but no further information recorded
	2017–07–24	Land	NA	9	10	NA	KW and PW sighted simultaneously but no further information recorded
	2017–07–27	Land	21	19–30	NA	Avoidance	PW approach KW, KW move away. Possible but unconfirmed high-speed avoidance (porpoising not confirmed). A single male KW is encircled by PW for a short time
	2017–07–30	Boat	51	NA	NA	Avoidance	PW approach KW, KW disappear from view. KW resighted and PW approach again at high speed, KW move away. Possible but unconfirmed high-speed avoidance (porpoising not confirmed)
	2018–07–10	Land	27	6–8	20	Avoidance	PW approach KW at high speed, KW move away fast. Possible but unconfirmed high-speed avoidance (porpoising not confirmed)
	2018–07–12	Boat	NA	12	20–28	No interaction	PW and KW sighted in the same area but no interaction observed. Two white-beaked dolphins (*Lagenorhynchus albirostris*) observed close to PW
	2018–07–14	Boat	94	16–37	30	Avoidance	Three groups of KW (16 individuals in total) at the start, after ~ 1 h several KW groups join (37 individuals in total). PW approach KW several times throughout, KW move away
	2018–07–24	Land	NA	NA	10–20	NA	KW and PW sighted simultaneously but KW only once and response unknown
	2019–06–23	Boat	46	20	30–40	Avoidance	PW approach KW at high speed twice, KW move away
	2019–06–30	Land	8	9–11	30–40	No response, avoidance	Two groups of KW. PW approach one KW group (4 individuals) at high speed. This KW group moves away. The other KW group (5–7 individuals) shows no response
	2019–07–17	Land	NA	5–6	30–40	No interaction	PW and KW sighted simultaneously but neither approaching the other. KW stay in the same area. PW are ~ 4 km from KW and move further away
	2019–07–22	Land	NA	8–10	20–50+	No interaction	PW and KW sighted simultaneously but neither approaching the other. KW stay in the same area. PW are ~ 5 km from KW and move further away
	2019–07–23	Land	286	5–8	11, 50+	No response	11 PW approach KW, KW stay in the same area, and PW eventually move away. Later, 50 + PW approach KW, KW stay in the same area, and PW eventually move away (Fig. 5). About 15 min later, the boat observes a high-speed avoidance in the same area but it was not possible to confirm if this involved the same individuals
		Boat	24	26–28	80–100	High-speed avoidance	Five groups of KW are present in the area. PW approach at high speed and one group of KW (6 individuals) moves away at high speed (porpoising, Figs. 3 and 5). The other groups stay behind. This observation is about 15 min after the observations from land end but it is unknown if this was the same group that had previously been approached by PW without responding in the observations from land
	2019–07–26	Boat	NA	5	30	Avoidance	PW approach, KW disappear from view
	2020–07–20	Boat	42	6	10+	No interaction	PW and KW sighted in the same area but no interaction observed
	2020–07–21	Land	139	19	40–45	Avoidance, high-speed avoidance	Four groups of KW and 2–3 groups of PW. One group of PW approach a group of KW (6 individuals), KW move away. The other two groups of PW approach different group of KW (4 individuals) at high speed, KW move away at high speed (porpoising)
		Boat	74	13	20–30	Avoidance	Same interaction as seen from land but high-speed avoidance not observed. Only 1–2 KW groups close enough to estimate group size and only one group of PW seen
	2020–07–24	Land	15	12–14	20–25	Avoidance	Two groups of KW. PW approach one group of KW (6 individuals) at high speed, KW move away
		Boat	24	9	20–30	Avoidance	Same interaction as seen from land but only one group of KW seen. The smaller group size observed from land might be because fewer individuals were clearly seen from that distance
	2020–07–25	Land	NA	6	5	NA	PW and KW sighted simultaneously but sight of KW lost shortly after as they went behind an island
Breiðafjörður	2017–08–02	Boat	18	15	150	High-speed avoidance	PW approach KW at high speed, KW move away at high speed (porpoising)
	2019–07–22	Boat	27	60	80	Avoidance	PW approach KW at high speed, KW move away
	2019–07–25	Boat	NA	40	40	High-speed avoidance	PW approach KW at high speed, KW move away at high speed (porpoising)
	2019–07–26	Boat	14	20	100	High-speed avoidance	PW approach KW at high speed, KW move away at high speed (porpoising)
	2020–07–19	Boat	NA	35	50	No interaction	PW and KW sighted in the same area but no interaction observed. A humpback whale (*Megaptera novaeangliae*) is observed surrounded by PW

**Fig. 3:**
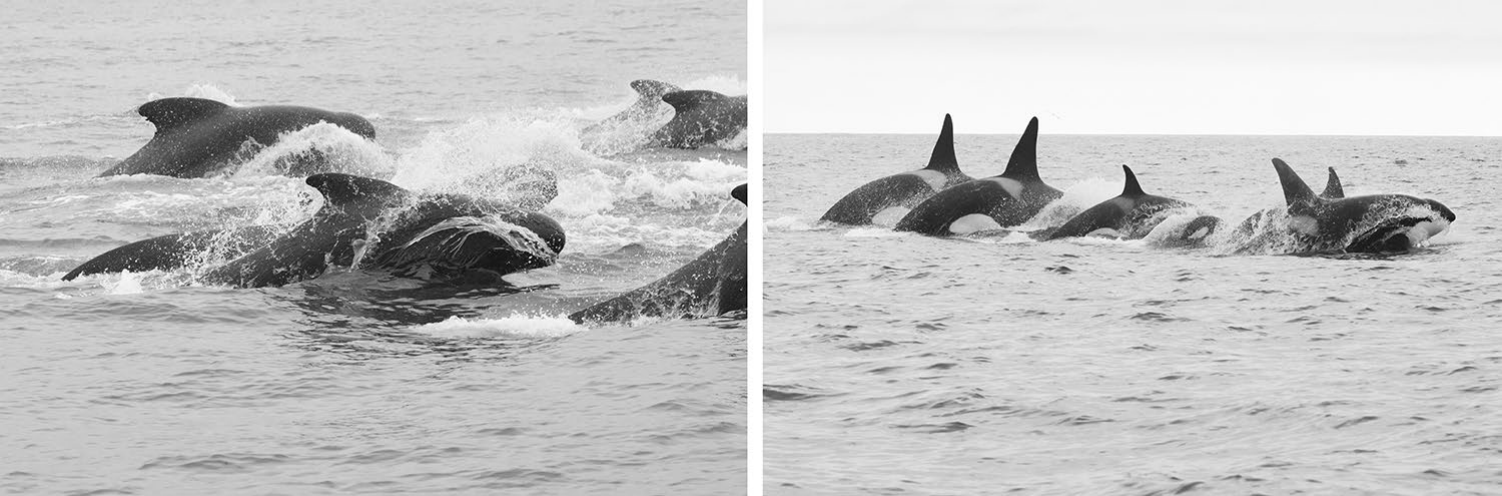
Pilot whales (left, photograph by Katarína Klementisová) and killer whales (right, photograph by Curt Hanson) porpoising during a high-speed avoidance observed in Vestmannaeyjar, Iceland, on 23 July 2019

In all interactions, pilot whales moved towards killer whales, causing the killer whales in most cases to move directly away from the pilot whales. Pilot whales typically approached killer whales at high speed, often porpoising, a behaviour used as an indicator of high-speed travel (Weihs 2002). The interactions could be divided into three categories, based on the behavioural response of the killer whales: no response, avoidance, and high-speed avoidance. In very few cases, the pilot whales approached and the killer whales showed no visible response (Table 2). Regular avoidance included instances where killer whales moved away from pilot whales at low to moderate speed, showed minor evasive behaviour, or disappeared from view for a few minutes and were then sighted some distance away. High-speed avoidance was assigned when killer whales were observed porpoising out of the water, with the pilot whales chasing the killer whales at high speed (Fig. 3, Video in Supplementary Material). Obvious aggressive physical contact between the two species was never observed. The interactions either ended with the pilot whales leaving the area or, in the case of high-speed avoidance behaviour, with both species slowing down and becoming less directional in their movements.

**Fig. 4:**
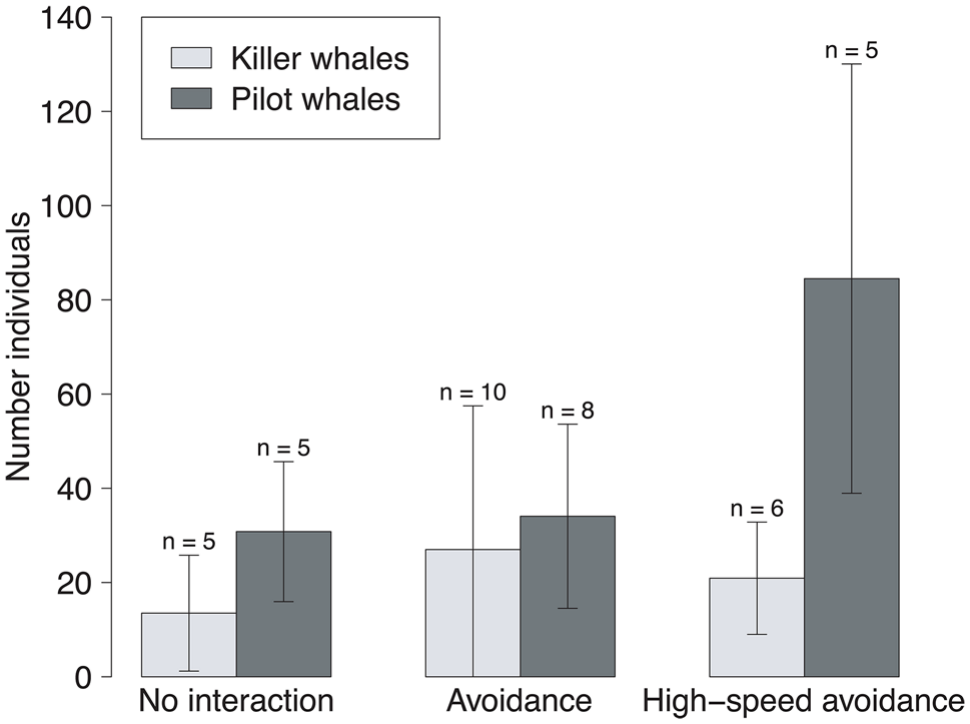
Group sizes during pilot whale approaches to killer whales, as a function of the behaviour of killer whales, and when no interaction was observed (i.e. both species were present in the same area simultaneously but no interaction was observed). Regular avoidance: the killer whales moved away from the approaching pilot whales at low to regular speed (i.e. an interaction); high-speed avoidance: the killer whales moved away from the approaching pilot whales and were observed ‘porpoising’ (i.e. an interaction). Interactions where killer whales showed no response were too few to be included. Bars represent the mean and error bars represent ± 1 standard deviation

The majority of interactions were categorised as regular avoidance (68.0%), but this should be considered a maximum estimate as the absence of porpoising was not always confirmed (Table 2). High-speed avoidance was confirmed in 28% of interactions. Pilot whale group size differed between regular avoidance, high-speed avoidance, and when no interaction was observed (Kruskal–Wallis test, *x*^2^(2) = 8.06, *p* < 0.05, Fig. 4). No interaction reflected times when both species were present in the same area simultaneously but there was no interaction between them. Observations of pilot whale approaches where killer whales showed no response (i.e. an interaction with no response) were too few to include in the comparison (*n* = 1). Pilot whale group sizes were highest during high-speed avoidance (85 ± 46, range 40–150, *n* = 5) compared to regular avoidance (34 ± 20, range 20–80, *n* = 8) and no interaction (31 ± 15, range 10–50, *n* = 5) but due to the small sample sizes, no further tests were performed. There was no evidence that the group size of killer whales varied across the three categories (Kruskal–Wallis, *x*^2^(2) = 1.09, *p* > 0.5, Fig. 4), although group sizes were largest for regular avoidance. However, usually several groups of killer whales were in the area and it was not always clearly recorded in the field notes whether all or only some groups responded. We could therefore only compare the total number of individuals observed.

### Examples of interactions between pilot and killer whales

**Fig. 5:**
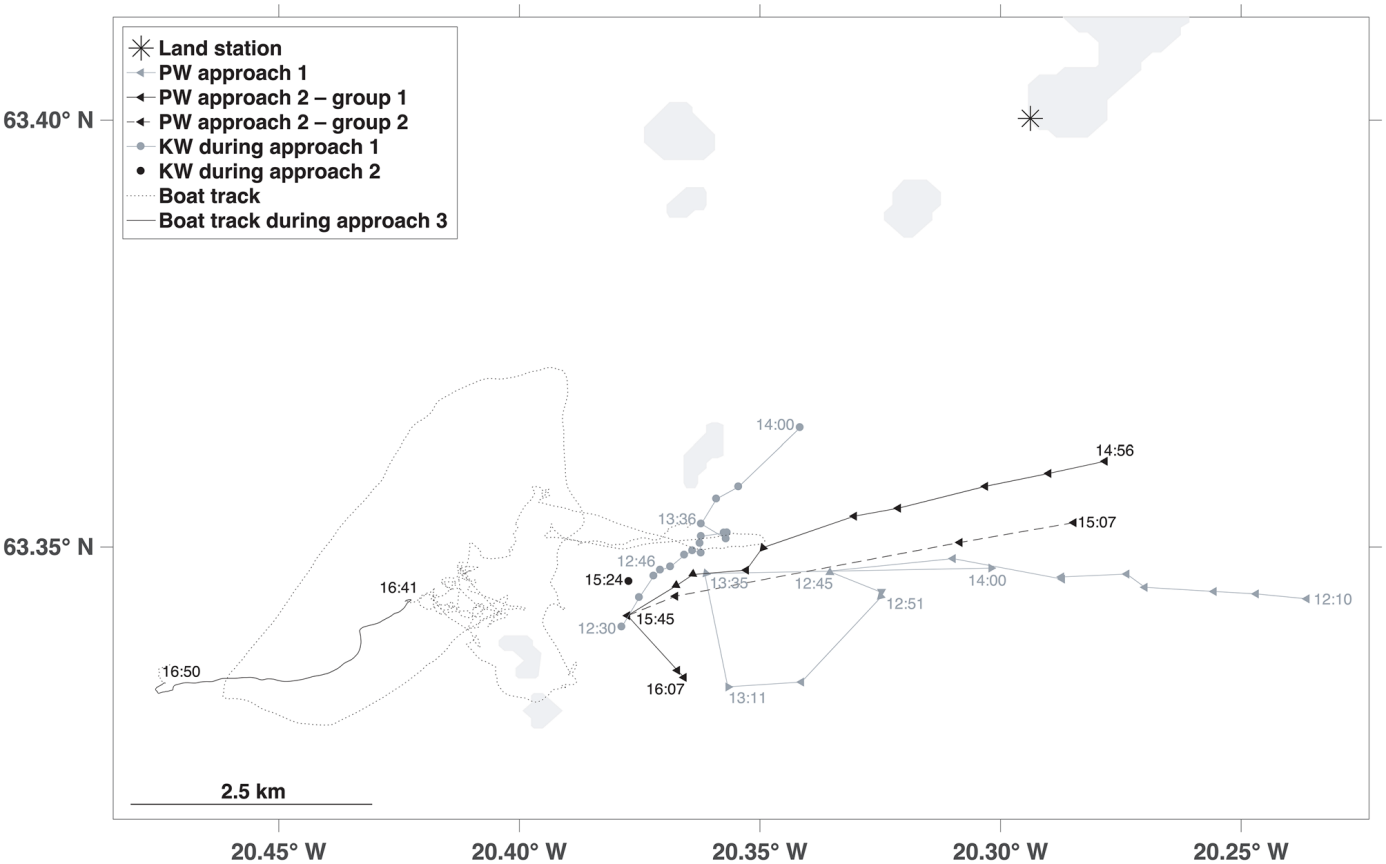
Example observation of interactions between pilot whales (PW) and killer whales (KW) in Vestmannaeyjar, Iceland, on 23 July 2019. Approaches 1 and 2 were observed from land and tracking data were collected with a theodolite. Approach 3 was observed from the boat. The boat closely followed the killer whales and thus the boat track can be considered the approximate track of the killer whales

Examples with movement tracks of three subsequent interactions can be seen in Fig. 5 and are briefly described here. These visual observations were collected on 23rd July 2019 in the Vestmannaeyjar archipelago and were made by boat, or from land with binoculars and a theodolite. Throughout the day, several groups of killer and pilot whales were observed. Visual observation of killer whales from land was obscured by islands during the second and third interaction events, but the boat was in proximity of killer whales throughout the entire observation period (10:51–17:03 UTC).

During the first approach, the pilot whales were only observed from the land station and no clear response of the killer whales was observed. At 11:29, 2–3 subgroups of pilot whales with 11 individuals in total were sighted. The groups were noted to merge and split several times while travelling fast towards the killer whales. Between 12:30 and 12:46, five killer whales were seen near one of the islands. At 12:45, the pilot whales made a sharp turn and started heading away from the killer whales. The pilot whales split and changed direction several times and then all started moving southwest at 12:51 and again towards the killer whales at 13:11. The killer whales changed direction frequently but stayed in the same area (‘milling’). At 13:35, the pilot whales were very close to the killer whales and changed direction a few times but then stopped travelling. The killer whales were logging at the surface. The pilot whales were not seen again until 14:00 when they were heading away from the killer whales.

The second approach was also observed from land and began at 14:45 when a group of 50+ pilot whales was sighted coming from the area where the previous group of pilot whales was last seen. Five killer whales were seen at 14:46 feeding close to one of the islands. At 14:50, the pilot whales split into two groups, both of which started approaching the killer whales at high speed and tracking of the two groups began at 14:56 and 15:07. At 15:24, one position measurement could be taken of the killer whales before they disappeared from view behind an island. Soon after (at 15:27), one of the two groups of pilot whales appeared to stop for a short time, and then at 15:36, the two subgroups merged. At 15:51, a group of eight killer whales was sighted close to the island but no coordinates could be measured. The pilot whales started to head away from the killer whales and were last seen at 16:15. As the killer whales were located close to an island that obstructed the view during parts of the approach, it is unclear whether they actively avoided the pilot whales.

The last approach by pilot whales was observed from the boat only. Five groups of killer whales with 26–28 individuals in total were observed from the boat between 16:01 and 16:36. At 16:35, 80–100 pilot whales were seen approaching from the east. A group of killer whales initially turned towards the pilot whales but then turned away, and at 16:41, the pilot whales were pursuing this group of six killer whales at high speed, with both species porpoising (Fig. 3, Video in Supplementary Material). The boat closely followed the killer whales for a short period (Fig. 5), allowing us to estimate a minimum swimming speed of at least 8 knots (15 km/h) and a maximum of 13.5 knots (25 km/h). At 16:50, the distance between the two species increased and both were slowing down. At this point, it is noted that several killer whales had stayed behind and had not been part of the group showing high-speed avoidance.

## Discussion

This study provides the first evidence of interspecific interactions between pilot whales and killer whales in Iceland, in regions where both species co-occur seasonally. The interactions appeared to be antagonistic, as suggested by previous studies in Spain and Norway, but in our study, interspecific interactions varied in intensity and not all close encounters of the two species led to observable interactions. These interactions are therefore more complex and widespread in the North Atlantic than previously thought. We observed changes in pilot whale occurrence along the Icelandic coast, particularly in the south where sightings appear to have increased over the study period, whereas killer whale occurrence did not change noticeably. The occurrence of both species overlapped spatially, with the south (Vestmannaeyjar) and west (Breiðafjörður) of Iceland recording regular sightings, and to a lesser degree also temporally, with both species regularly occurring in summer, especially in the south. In the west, killer whales were sighed more often in winter to early summer and pilot whales in late summer, so their temporal overlap was lower than in the south of Iceland.

Estimates for pilot whale population size in offshore areas of the Northeast Atlantic between 1987 and 2015 showed no significant population growth or decline (Pike et al. 2019); thus, it appears that changes in population size are unlikely to have led to the apparent increase in pilot whale sightings in coastal waters off south Iceland. The offshore occurrence of pilot whales was consistently highest in the waters southwest of Iceland and along the continental edge in south Iceland (Buckland et al. 1993; Pike et al. 2019), areas that are geographically close to most of the coastal waters that had high pilot whale occurrence in this study. Similarly, strandings were also reported most often in the southwest, west, and northwest of Iceland (Sigurjónsson et al. 1993). Therefore, it is possible that rather than changes in population size, it is changes in distribution that may explain the apparent increase observed in this study, with pilot whales that generally inhabit offshore waters venturing inshore in some years.

A change in a predator’s distribution from offshore to inshore waters could be due to changes in prey availability (McComb-Turbitt et al. 2021). Pilot whales in the North Atlantic appear to feed predominantly on squid (Desportes and Mouritsen 1993; Sigurjónsson et al. 1993; Gannon et al. 1997; Santos et al. 2014). Squid undergo extensive migrations for feeding and spawning but their occurrence is highly irregular and likely influenced by environmental conditions (Jákupsstovu 2002; Hátún et al. 2009). In so-called squid years, large aggregations of squid appear around Iceland, the Faroe Islands, and off Norway, and are associated with higher catches of pilot whales around the Faroe Islands (Jákupsstovu 2002; Hátún et al. 2009). Similarly, catch numbers and stranding reports from Iceland suggest periods of higher occurrence of pilot whales could be linked to irregular migration of squid (Sæmundsson 1937, 1939; Sigurjónsson et al. 1993). Pilot whale sightings in Icelandic coastal waters in this study were only reported in June–September, which coincides with high abundance of squid and pilot whales around the Faroe Islands, particularly in years of high squid occurrence (Jákupsstovu 2002). Thus, the observed increase of occurrence of pilot whales around Iceland could be due to a period of high squid abundance in Icelandic coastal waters.

Alternatively, pilot whales could be following mackerel (*Scomber scombrus*), which appears to be an important prey of pilot whales elsewhere in the North Atlantic (Overholtz and Waring 1991; Abend and Smith 1997; Gannon et al. 1997). Mackerel abundance increased dramatically in Icelandic offshore and coastal waters between 2007 and 2014 and is still caught in Icelandic waters to date (Astthorsson et al. 2012; MFRI 2021a). Since pilot whales typically feed at depth, observing feeding behaviour and identifying prey species from surface observations is difficult (Baird et al. 2002; Visser et al. 2014). One of our observations in Breiðafjörður included a gull picking up a mackerel near a group of pilot whales apparently feeding but this was the only time a possible prey species was identified in this study. Finally, pilot whale distribution could also be influenced by climatic changes (van Weelden et al. 2021). Pilot whales in the western North Atlantic are shifting north at rates faster than their prey, likely showing a direct response to warming waters (Thorne and Nye 2021). Icelandic waters have been warming (based on sea surface temperature) over the last 20 years (Óskarsson et al. 2021), which could impact the distribution of highly mobile species, such as pilot whales. Data collection for our study was mostly opportunistic. This could have led to a bias in coverage of areas and time of year, as well as the individuals sampled, that was not possible to control for. In addition, coverage differed highly between locations, years, and seasons (Table 1 and Fig. 2). Thus, it is difficult to make conclusive inferences about changes over time. Systematic, long-term data collection on pilot whale occurrence and diet will be required to fully understand the patterns and the drivers of distribution in Iceland.

Killer whale occurrence did not appear to change throughout the study period but did show regional and seasonal variations. Killer whales were most frequently encountered in Vestmannaeyjar and Breiðafjörður, which are important herring grounds. Herring abundance in Iceland has declined since mackerel became abundant (MFRI 2021a, b) and the distribution of the two species often overlaps (Óskarsson et al. 2016). Opportunistic observations in Vestmannaeyjar and Breiðafjörður indicate the presence of mackerel in summer in some years. It is unknown to what extent killer whales in Iceland have taken advantage of the incoming mackerel, but in neighbouring regions of the North Atlantic, it is known to be an important prey, even for whales suspected to feed on herring as well (Luque et al. 2007; Foote et al. 2012; Nøttestad et al. 2014). Pilot whale stomach contents in Iceland and the Faroe Islands have not included herring or mackerel (Desportes and Mouritsen 1993; Sigurjónsson et al. 1993). Thus, pilot whales may occur most often in locations where killer whales are also frequently seen due to an overlap in the distribution of preferred prey (herring and squid or herring and mackerel). Alternatively, the possibility that both species are feeding on mackerel or herring, and therefore competing for resources, cannot be excluded at present. Given that the extent of temporal overlap of occurrence of pilot and killer whales in the south and west of Iceland is not identical, further research into the diet and habitat use of both species in both locations would be necessary to establish potential dietary overlap.

In the locations that had concurrent sightings of pilot whales and killer whales, interspecific interactions were common, particularly in south Iceland where their seasonal occurrence also overlapped to the greatest extent. These interactions appeared to be antagonistic, with killer whales often avoiding pilot whales and sometimes fleeing at high speed. Similar interactions have been previously reported from northern Norway and the Strait of Gibraltar, Spain (Stenersen and Similä 2004; de Stephanis et al. 2014). In our observations, not all sightings of the two species in the same area resulted in interactions, and high-speed avoidance with porpoising was only observed in 28% of interactions. On several days, pilot whales approached killer whales multiple times and killer whale responses to these approaches could vary from no response to avoidance or high-speed avoidance with observed porpoising. This indicates that these interactions are complex and it remains unclear what exactly triggers the avoidance by killer whales and, in particular, the high-speed response. Our results suggest that responses by killer whales could be related to the group size of pilot whales, with larger pilot whale groups leading to high-speed avoidance. However, as data were collected ad libitum and pilot whale group sizes are difficult to estimate (Pike et al. 2019), we cannot rule out that the increased speed during these events led to more whales being visible at the surface, which could have inflated estimates. Similarly, it was not always clear whether all killer whale groups present responded in the same manner and thus what was the number of individuals in the responding group(s). This emphasises the need to systematically study these interactions, with a standardised data collection protocol, in the future. Previous studies have suggested that acoustic cues could play a role in triggering the behavioural response of killer whales (Stenersen and Similä 2004). Thus, further studies on the role of acoustic behaviour during interactions could help elucidate the triggers for different types of responses by killer whales.

Interactions between pilot whales and killer whales have previously been suggested to be driven by competition or predator deterrence (Stenersen and Similä 2004; Curé et al. 2012, 2019; de Stephanis et al. 2014). Competition could occur, for example, for habitat, foraging areas, or prey. As mentioned above, there is a possibility for spatial overlap in prey species or dietary overlap between pilot whales and killer whales in Icelandic waters. In this study, pilot whales were not often observed displaying behaviour consistent with feeding at depth (i.e. milling at the surface and spreading out; Visser et al. 2014). However, killer whales were often observed feeding when the pilot whales approached in many of the interactions. Overall, feeding was the most common behavioural state that was observed in killer whales in this study and it remains unclear whether pilot whales preferentially approached feeding killer whales.

Stable isotope analyses from the Strait of Gibraltar suggested little dietary overlap and no predation of either species on the other (de Stephanis et al. 2014). Playback experiments further indicate that simulated killer whale presence does not induce foraging behaviour in pilot whales and that pilot whales can distinguish between the sounds of familiar fish-eating and unfamiliar mammal-eating killer whales, perceiving the former as a lower threat than the latter (Curé et al. 2019). Thus, the observed interactions between pilot and killer whales were suggested to be an active intimidation behaviour against a perceived low-threat predator (de Stephanis et al. 2014; Curé et al. 2019). Killer whale predation on short- or long-finned pilot whales has been reported occasionally (Jefferson et al. 1991; Nishiwaki and Handa 1958), including a potential predation event in Iceland (Donovan and Gunnlaugsson 1989), but interactions without aggressive behaviour or mixed aggregations of both species have also been reported (Jefferson et al. 1991). In Iceland, reports of predation by killer whales on cetacean species are infrequent (Samarra et al. 2018). Thus, pilot whales in Iceland may perceive killer whales as a low-threat predator and exhibit a mobbing response, but competition between both species cannot be excluded until further information on their habitat use, behaviour, and feeding ecology is gathered.

Killer whales commonly abandoned feeding events when avoiding pilot whales; thus, the occurrence of these interspecific interactions may carry energetic consequences for killer whales. Reduction in foraging effort can have considerable energetic impacts (Williams et al. 2006), which could increase due to energy expenditure in high-speed avoidance (Allen et al. 2022). Therefore, more detailed research into pilot-killer whale interactions, including their ecological and evolutionary drivers and energetic impacts, would be highly relevant to inform conservation management for both species.

## Supplementary Information

Below is the link to the electronic supplementary material.

Supplementary file1 (XLSX 35.7 KB)

Supplementary file2 (MP4 46180 KB)

## Data Availability

Data provided as supplementary material.
